# Can Serum Gamma-Glutamyl Transferase Predict All-Cause Mortality in Hypertensive Patients?

**DOI:** 10.7759/cureus.68247

**Published:** 2024-08-30

**Authors:** Ryuichi Kawamoto, Asuka Kikuchi, Daisuke Ninomiya

**Affiliations:** 1 Department of Community Medicine, Ehime University Graduate School of Medicine, Seiyo, JPN; 2 Department of Community Medicine, Ehime University Graduate School of Medicine, Toon, JPN

**Keywords:** japanese, community-dwelling persons, hypertension, mortality marker, serum gamma-glutamyl transferase

## Abstract

Introduction

In this investigation, the focus was on exploring the connection between serum gamma-glutamyl transferase (GGT), a vital element that influences health and mortality in relation to aging, and all-cause mortality. The study was conducted using a follow-up approach at eight- and 20-year intervals.

Methods

The study involved 1,101 female participants, with an average age of 69 years (± 9), and 916 male participants, with an average age of 67 years (± 11), who were all diagnosed with hypertension. These individuals were drawn from the Nomura cohort study, which consisted of two separate cohorts: the initial cohort established in 2002 and the subsequent cohort in 2014. We used a Cox proportional hazards model to calculate the hazard ratios (HRs), adjusted for multiple variables, for mortality risk from the initial health examination until the conclusion of the follow-up periods.

Results

The study followed the participants for a median period of 13.9 years (interquartile range: 8.5-20.2 years). During this follow-up period, 716 deaths were recorded in this population (360 in men and 356 in women), resulting in a mortality rate of 25.5 deaths per 1,000 person-years. Male participants categorized with serum GGT levels ranging from 42 to 86 IU/L showed a 64% increased risk of all-cause mortality (HR: 1.64; 95% confidence interval (CI): 1.11-2.40), while those with GGT levels of 87 IU/L or higher exhibited a 93% elevated risk (HR: 1.93; 95% CI: 1.18-3.16) compared to individuals with GGT levels below 19 IU/L. The association between higher GGT levels and increased all-cause mortality was more evident in men than in women, with a significant interaction between gender and baseline serum GGT (p = 0.020).

Conclusions

Our findings suggest a notable correlation between irregular GGT levels and the overall mortality rate among Japanese individuals with hypertension living in community settings. Notably, especially in older males, GGT activity turns out to be a critical biomarker for predicting long-term survival.

## Introduction

The serum gamma-glutamyl transferase (GGT) enzyme is frequently employed as a clinical indicator for liver or biliary tract disorders, as well as alcohol intake. Research has indicated that there are additional factors linked to serum GGT levels. GGT is not limited to the liver; it is also present in various other organs, such as the lung, pancreas, kidney, and vascular endothelium. Serum GGT activity is believed to indicate changes in oxidative stress, potentially through its direct involvement in generating reactive oxygen species and indirectly by facilitating the transportation of glutathione precursors into cells [1]. As a result, numerous large-scale epidemiological studies have established a correlation between elevated GGT levels and cardiovascular disease (CVD) [2,3]. Furthermore, studies have indicated that GGT is a marker for other concurrent risk factors, such as obesity, insulin resistance [4], fatty liver (e.g., metabolic-associated fatty liver disease) [5], diabetes [6], hypertension [7], dyslipidemia, and metabolic syndrome [8,9]. Serum GGT levels have also been identified as a prognostic factor for overall population mortality. GGT levels have been associated with higher risks of all-cause and CVD mortality, even when they are within the normal range [10]. This is true even when confounding variables are taken into consideration.

Hypertension is associated with an increased risk of death from all causes, mainly from heart and blood vessel disorders. It is unlikely that some of the remaining associations are causal [11]. To better understand the relationships between hypertension and less common causes of death and to establish causality for various outcomes, more research is required. The association between GGT levels and the prevalence of all-cause mortality in community-dwelling individuals with hypertension is not well understood at this time.

In this study, we looked at the distribution of GGT levels and how they related to mortality from all causes in hypertensive patients who lived in the community. Utilizing cohort data, we also looked into age as a potential risk factor.

## Materials and methods

Study design and participants

**Figure 1:**
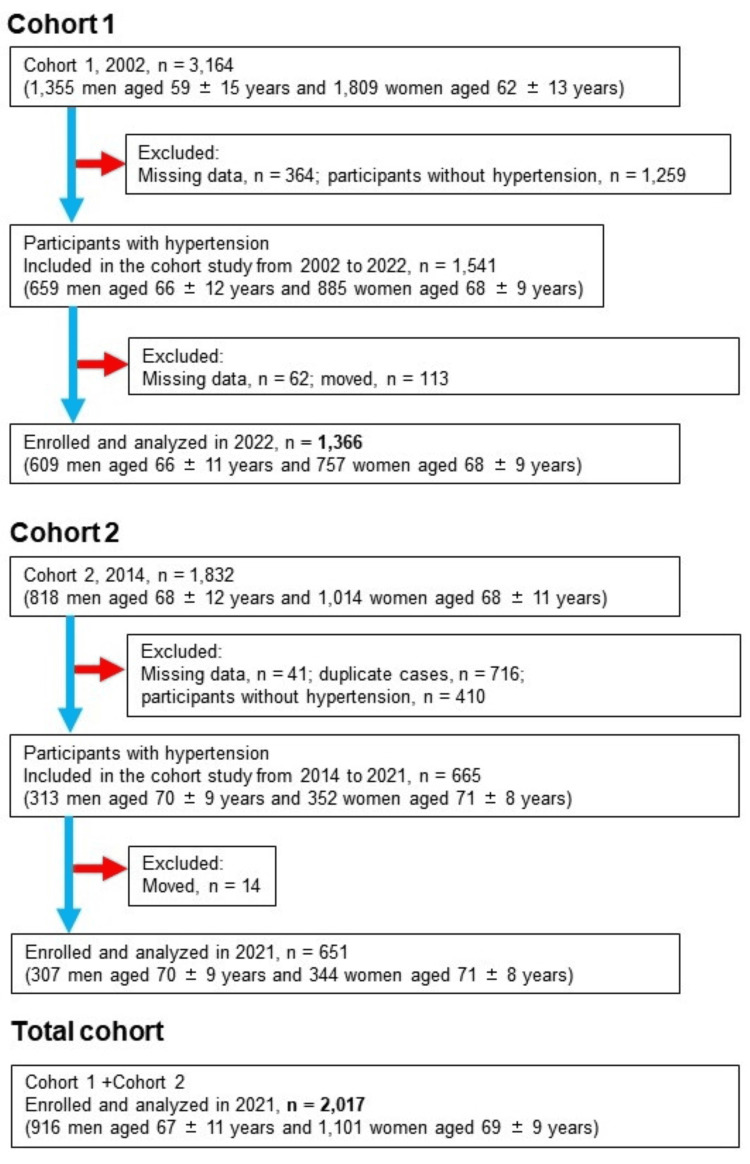
Flowchart of participants

A prospective cohort analysis was conducted in two stages, in 2002 and 2014, within the framework of the Nomura study [12]. The research targeted individuals primarily residing in rural areas of Ehime Prefecture who had participated in community-based annual health screenings. The primary focus of the research was on individuals diagnosed with hypertension, defined by a systolic blood pressure (SBP) of 140 mmHg or higher, a diastolic blood pressure (DBP) of 90 mmHg or higher, or those receiving antihypertensive medication. The initial cohort comprised 1,541 participants, while the second cohort consisted of 665 participants, all aged between 23 and 89 years. Among these, 1,366 individuals from the initial cohort and 651 from the second cohort underwent initial physical examinations and were subsequently enrolled in the follow-up study. The overlapping cases between the first cohort and the second cohort were identified using the health checkup IDs. Data from both cohorts, totaling 2,017 participants, were meticulously analyzed (Figure 1). A self-administered questionnaire was utilized to collect data on the participants' habits, medical background, present health condition, and medication intake. For the first cohort, follow-up surveys were conducted at intervals of 20 years; for the second cohort, the follow-up surveys were conducted at intervals of eight years. The survival status of participants was verified through Japan's Basic Resident Register. The study protocols were subjected to a thorough review and subsequently approved by the Institutional Review Board (IRB) at Ehime University Hospital, under approval number 1903018. Prior to their involvement in the study, all participants consented in writing after being provided with comprehensive information regarding the study.

Evaluation of risk factors

The research involved assessing the weight and height of participants. BMI was determined by dividing weight in kilograms by the square of height in meters. Pack-years, which are computed by multiplying the total number of years smoked by the average number of packs smoked per day, were used to evaluate smoking habits. There were four categories for smoking groups: light smokers (less than 20 pack-years), heavy smokers (more than 20 pack-years), non-smokers, and former smokers. Sake units, or 22.9 g of ethanol, were used to measure the amount of alcohol consumed each day [13]. The categories for alcohol consumption patterns were as follows: abstainers, occasional drinkers (less than one unit per day), light daily drinkers (one to two units per day), and heavy daily drinkers (two to three units per day). Not a single participant consumed more than three units daily.

An automatic sphygmomanometer was used to measure SBP and DBP. Participants were asked to sit quietly for at least five minutes prior to the readings being taken. The right upper arm of each participant was fitted with a cuff. Additionally, participants underwent blood tests to evaluate various parameters, including triglycerides (TG), serum uric acid (SUA), blood glucose (BG), creatinine (Cr), high-density lipoprotein cholesterol (HDL-C), low-density lipoprotein cholesterol (LDL-C), GGT, alanine transaminase (ALT), and aspartate transaminase (AST). Participants were required to fast for the entire night before these tests. An automated analyzer (TBA-c16000, TOSHIBA, Tokyo, Japan) was used to measure the levels of GGT in serum; the assay's intra-assay coefficients of variation ranged from 0.87% to 2.11%. To calculate the estimated glomerular filtration rate (eGFR), we utilized the Chronic Kidney Disease Epidemiology Collaboration (CKD-EPI) equation, adjusting it with coefficients specific to the Japanese population. For males with serum Cr levels of 0.9 mg/dL or lower, the formula was 141 × (Cr/0.9)^-0.411 × 0.993^age × 0.813. If the Cr concentration exceeded 0.9 mg/dL, the formula changed to 141 × (Cr/0.9)^-1.209 × 0.993^age × 0.813. Similarly, for females with Cr levels of 0.7 mg/dL or lower, the equation was 144 × (Cr/0.7)^-0.329 × 0.993^age × 0.813. If the Cr level surpassed 0.7 mg/dL, the equation became 144 × (Cr/0.7)^-1.209 × 0.993^age × 0.813 [14].

The participants were categorized according to predetermined standards. Hypertriglyceridemia was defined as having TG levels ≥ 150 mg/dL. Hypo-HDL cholesterolemia was identified in individuals with HDL-C levels of ≤ 40 mg/dL. Participants were categorized as having hyperlipidemia if their LDL-C levels were equal to or greater than 140 mg/dL or if they were using antidyslipidemic medication. Diabetes was identified in participants with BG levels ≥ 126 mg/dL who were also receiving antidiabetic medication. Hyperuricemia was characterized as SUA levels ≥ 7.0 mg/dL, and those using SUA-lowering medication were placed in this category. Finally, an eGFR of < 60 mL/min/1.73 m^2^ was used as a CKD marker. Patients were categorized as having CVD if they had received diagnoses of conditions such as ischemic heart disease, peripheral vascular disease, or ischemic stroke.

Statistical analysis

The data were subjected to statistical analysis using IBM SPSS Statistics (version 27.0; SPSS, Armonk, NY). Continuous variables were presented as the mean ± standard deviation (SD). For variables that deviated from a normal distribution (such as TG, BG, GGT, ALT, and AST), median and interquartile range values were provided. Log-transformed values were used for parameters with non-normal distributions in all analyses. Participants were categorized into different groups based on the standard deviation of their GGT levels. For males, these groups were classified as low (< 20 IU/L), medium (20-41 IU/L), high (42-86 IU/L), or very high (≥ 87 IU/L), while for females, the categories were low (< 13 IU/L), medium (13-21 IU/L), high (22-36 IU/L), or very high (≥ 37 IU/L). Chi-squared tests were employed to compare categorical variables, while analysis of variance (ANOVA) was utilized for normally distributed continuous variables. Each baseline characteristic was individually analyzed using the Cox proportional hazards model to identify significant confounding factors, which were then included as covariates. Subsequently, a multivariable analysis was performed using the forced-entry method in the Cox proportional hazards model, with age as the primary time variable. Subgroup analyses were conducted to evaluate the consistency of the identified association between GGT levels and all-cause mortality. Interactions between GGT groups and subgroup variables were assessed using likelihood ratio tests. interaction analysis was conducted to evaluate the impact of the variable, with adjustments made for all confounding factors (excluding the impact variable). All p-values were computed as two-tailed, and statistical significance was defined as p < 0.05.

## Results

The baseline characteristics of participants with hypertension stratified according to their baseline serum GGT levels

The sample comprised 2,017 participants, with males constituting 45.4% of the total. The average age for males was 67 ± 11 years, whereas for females, it was 69 ± 9 years. The median (interquartile range) follow-up period was 5,200 (3,094-7,381) days. During the follow-up surveys, 716 deaths were recorded, representing 35.5% of all participants. Out of these, 360 were male (39.3% of all males), and 356 were female (32.3% of all females). Table 1 displays the baseline characteristics of the participants, categorized based on baseline serum GGT levels. With an increase in the GGT category, there was a significant increase in BMI, smoking and drinking habits, SBP, DBP, prevalence of hypertriglyceridemia, TG, eGFR, prevalence of hyperuricemia, and SUA, ALT, and AST levels. However, we observed no association between GGT categories and history of CVD, HDL-C, prevalence of hypo-HDL cholesterolemia, hyper-LDL cholesterolemia, diabetes, or CKD.

**Table 1:** Baseline characteristics of participants with hypertension, stratified by the baseline serum gamma-glutamyl transferase (GGT) category The data are expressed as mean ± standard deviation. However, triglycerides, blood glucose, gamma-glutamyl transferase, alanine transaminase, and aspartate transaminase data exhibited skewness and are therefore presented as median (interquartile range) values and log-transformed for analysis. * P-values are derived from ANOVA tests for continuous variables or χ^2^ tests for categorical variables. Significant values (p < 0.05) are presented in bold.

	Baseline serum GGT categories																
Men	< 20				20–41				42–86				≥ 87 IU/L				
Women	< 13				13–21				22–36				≥ 37				
Baseline characteristics, n = 2,017	n = 242				n = 914				n = 577				n = 284				p-value *
Gender (male), n (%)	124 (51.2)				394 (43.1)				253 (43.8)				145 (51.1)				0.023
Age (years)	74 ± 8				70 ± 9				66 ± 10				63 ± 10				< 0.001
Body mass index (kg/m^2^)	22.4 ± 2.5				23.6 ± 3.1				24.4 ± 3.3				24.9 ± 3.5				< 0.001
Smoking habits (never/past/light/heavy) (%)	68.6	18.6	6.6	6.2	70.4	14.8	5.9	9.0	65.9	13.7	6.4	14.0	58.8	14.1	7.4	19.7	< 0.001
Drinking habits (never/occasional/light/heavy) (%)	69.8	19.8	6.2	4.1	58.6	24.1	12.7	4.6	52.5	21.1	13.9	12.5	39.4	18.0	16.2	26.4	< 0.001
History of cardiovascular disease, n (%)	30 (12.4)				79 (8.6)				74 (12.8)				32 (11.3)				0.055
Hypertension, n (%)	------				------				------				------				------
Systolic blood pressure (mmHg)	147 ± 17				151 ± 16				151 ± 17				152 ± 17				< 0.001
Diastolic blood pressure (mmHg)	81 ± 10				85 ± 10				87 ± 10				89 ± 10				< 0.001
Use of antihypertensive medication, n (%)	141 (58.3)				508 (55.6)				314 (54.4)				148 (62.1)				0.533
Hypertriglyceridemia, n (%)	19 (7.9)				136 (14.9)				141 (24.4)				110 (38.7)				< 0.001
Triglycerides (mg/dL)	81 (61–103)				92 (71–125)				108 (78–148)				121 (84–182)				< 0.001
Hypo-HDL-cholesterolemia, n (%)	7 (2.9)				29 (3.2)				28 (4.9)				8 (2.8)				0.265
HDL cholesterol (mg/dL)	63 ± 14				62 ± 15				62 ± 16				63 ± 17				0.790
Hyper-LDL-cholesterolemia, n (%)	82 (33.9)				315 (34.5)				215 (37.3)				95 (33.5)				0.606
LDL cholesterol (mg/dL)	118 ± 27				120 ± 29				121 ± 31				113 ± 35				0.004
Use of lipid-lowering medication, n (%)	35 (14.5)				112 (12.3)				83 (14.4)				33 (11.6)				0.502
Diabetes, n (%)	47 (19.4)				135 (14.8)				101 (17.5)				52 (18.3)				0.209
Blood glucose (mg/dL)	111 (96–123)				103 (93–117)				104 (95–120)				105 (94–118)				0.002
Use of anti-diabetic medication, n (%)	27 (11.2)				92 (10.1)				76 (13.2)				39 (13.7)				0.188
Chronic kidney disease, n (%)	39 (16.1)				134 (14.7)				68 (11.8)				34 (12.0)				0.219
eGFR (mL/min/1.73 m^2^)	71.0 ± 14.8				73.7 ± 14.8				75.4 ± 15.6				78.3 ± 17.1				< 0.001
Hyperuricemia, n (%)	29 (12.0)				118 (12.9)				110 (19.1)				71 (25.0)				< 0.001
Serum uric acid (mg/dL)	5.0 ± 1.3				5.1 ± 1.4				5.3 ± 1.4				5.9 ± 1.6				< 0.001
Serum uric acid lowering medication, n (%)	7 (2.9)				50 (5.5)				45 (7.8)				19 (6.7)				0.043
Gamma glutamyl transferase (IU/L)	12 (11–16)				20 (16–27)				33 (25–53)				96 (50–155)				< 0.001
Alanine transaminase (IU/L)	14 (12–18)				16 (13–27)				20 (16–26)				29 (21–41)				< 0.001
Aspartate transaminase (IU/L)	22 (20–26)				22 (19–27)				24 (21–28)				30 (23–40)				<0.001

Adjusted survival curves for baseline serum GGT categories

**Figure 2:**
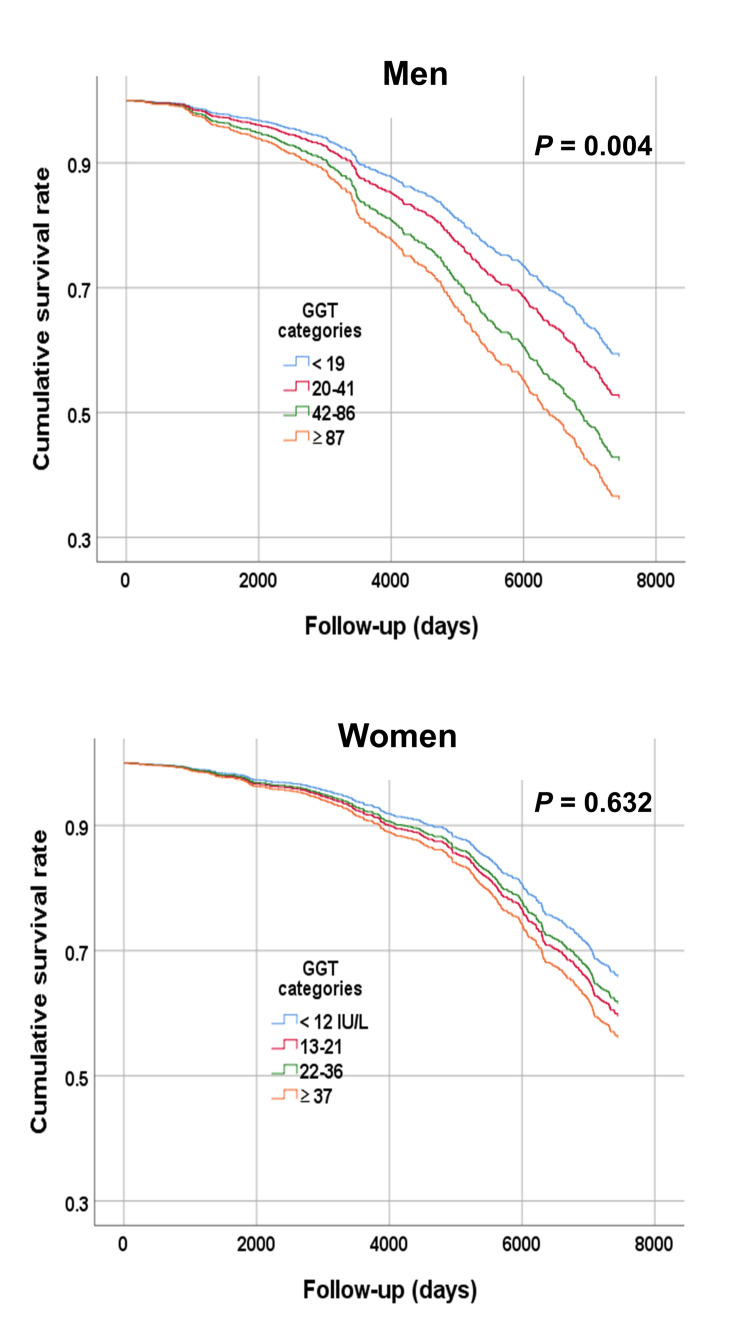
Analysis of associations between serum gamma-glutamyl transferase (GGT) categories and all-cause mortality during follow-up Participants are classified according to gender and categorized as having low (< 20 IU/L), medium (20–41 IU/L), high (42–86 IU/L), or very high (≥ 87 IU/L) gamma-glutamyl transferase (GGT) levels if they are male, or low (< 13 IU/L), medium (13–21 IU/L), high (22–36 IU/L), or very high (≥ 37 IU/L) if they are female. Among men, elevated GGT levels were significantly linked to a reduced cumulative survival rate (p = 0.004).

Figure 2 displays adjusted survival curves depicting both survival days and cumulative survival rates. These curves were generated to explore the relationships between the four GGT categories and all-cause mortality. Among male participants, the analysis indicated a significantly diminished cumulative survival rate for individuals with GGT levels of ≥ 87 IU/L (hazard ratio (HR), 1.83; 95% confidence interval (CI): 1.21-2.76), and 42-86 IU/L (HR, 1.56; 95% CI: 1.10-2.21) compared to those with the lowest GGT levels (p = 0.004).

Hazard ratios for all-cause mortality based on baseline serum GGT categories stratified by gender

The median duration of follow-up was 13.9 years, with an interquartile range of 8.5-20.2 years. During this timeframe, there were a total of 716 deaths (360 in men and 356 in women) in the overall population, yielding a mortality rate of 25.5 deaths per 1,000 person-years. Table 2 illustrates that, among male participants, those falling into the GGT categories of 42-86 IU/L (HR: 1.64; 95% CI: 1.11-2.40) and ≥ 87 IU/L (HR: 1.93; 95% CI: 1.18-3.16) displayed an elevated risk of all-cause mortality compared to those in the reference category (< 20 IU/L). This analysis was adjusted for various factors including age, gender, BMI, smoking status, alcohol consumption, hypertriglyceridemia, hypo-HDL cholesterolemia, hyper-LDL cholesterolemia, diabetes, CKD, hyperuricemia, ALT, and AST. As a supplemental table, the Appendix (Table 4) shows the HR (95% CI) for baseline GGT classification for all-cause mortality in participants without hypertension. Only in men was there a significant increase in mortality in the GTT category of ≥ 87 IU/L, but the trend between categories was not significant.

**Table 2:** Hazard ratios and 95% confidence intervals of baseline serum gamma-glutamyl transferase (GGT) categories for all-cause mortality by gender Model 1 was adjusted for age and gender. Model 2 included additional adjustments for body mass index, smoking status, alcohol consumption, and history of cardiovascular disease, in addition to the covariates in Model 1. Model 3 further included adjustments for hypertriglyceridemia, hypo-HDL cholesterolemia, hyper-LDL cholesterolemia, diabetes, chronic kidney disease, hyperuricemia, alanine aminotransferase, and aspartate aminotransferase, in addition to the covariates in Model 2. Significant values (p < 0.05) are presented in bold.

	Baseline serum GGT categories						
Men (n = 916)	< 20	20–41	42–86	≥ 87 IU/L			
	n = 124	n = 394	n = 253	n = 145		p for trend	
Prevalence of death (%)	51 (41.1)	161 (40.9)	96 (37.9)	52 (35.9)		0.684	
Model 1	Reference	1.14 (0.83–1.56)	1.56 (1.10–2.21)	1.83 (1.21–2.76)		0.004	
Model 2	Reference	1.20 (0.87–1.66)	1.58 (1.09–2.30)	1.92 (1.22–3.02)		0.014	
Model 3	Reference	1.22 (0.88–1.70)	1.64 (1.11–2.40)	1.93 (1.18–3.16)		0.022	
Women (n = 1,101)	< 13	13–21	22–36	≥ 37 IU/L			
	n = 118	n = 520	n = 324	n = 139			
Prevalence of death (%)	26 (22.0)	184 (35.4)	105 (32.4)	41 (29.5)		0.038	
Model 1	Reference	1.24 (0.82–1.88)	1.18 (0.76–1.83)	1.34 (0.81–2.21)		0.688	
Model 2	Reference	1.22 (0.84–1.86)	1.15 (0.74–1.78)	1.33 (0.80–2.20)		0.688	
Model 3	Reference	1.24 (0.82–1.89)	1.16 (0.74–1.83)	1.41 (0.81–2.45)		0.597	
Overall (n = 2,017)							
Men	< 20	20–41	42–86	≥ 87 IU/L			
Women	< 13	13–21	22–36	≥ 37 IU/L			
	n = 242	n = 914	n =577	n = 284			
Prevalence of death (%)	77(31.8)	345 (37.7)	201 (34.8)	93 (32.7)		0.212	
Model 1	Reference	1.20 (0.95–1.57)	1.39 (1.06–1.82)	1.64 (1.19–2.24)		0.011	
Model 2	Reference	1.24 (0.96–1.60)	1.38 (1.04–1.82)	1.65 (1.18–2.29)		0.023	
Model 3	Reference	1.27 (0.98–1.65)	1.44 (1.08–1.92)	1.75 (1.22–2.52)		0.020	

Hazard ratios for all-cause mortality based on baseline serum GGT categories in the sub-analysis

Table 3 provides a breakdown of participants based on the two cohorts (cohort 1 and cohort 2), gender (men and women), age (< 65 and ≥ 65 years), BMI (< 25 kg/m^2^ and ≥ 25 kg/m^2^), history of CVD (absence and presence), antihypertensive medication (absence and presence), and time until death (< 1,095 days or ≥ 1,095 days). Consistent with our previous findings, elevated GGT levels within the normal range were associated with a higher risk of all-cause mortality. This connection was notably pronounced in men aged ≥ 65 years and in individuals with a BMI < 25 kg/m^2^ and no prior history of CVD. Moreover, the influence of elevated GGT levels on mortality exhibited greater significance in men compared to women, with a significant interaction observed between gender and baseline serum GGT concerning all-cause mortality (p= 0.020).

**Table 3:** Hazard ratios and 95% confidence intervals of baseline serum gamma-glutamyl transferase (GGT) categories for all-cause mortality by sub-analysis HR, hazard ratio; CI, confidence interval. The multivariate-adjusted hazard ratio (HR) was calculated after accounting for age and gender, as well as other factors including body mass index, smoking status, alcohol consumption, history of cardiovascular disease, hypertriglyceridemia, hypo-HDL cholesterolemia, hyper-LDL cholesterolemia, diabetes, chronic kidney disease, hyperuricemia, alanine aminotransferase, and aspartate aminotransferase. Significant values (p < 0.05) are presented in bold.

	Multivariable-adjusted HR (95% CI)											
Characteristics, n = 2,017	Baseline serum GGT categories											
Men	< 20		20–41		42–86		≥ 87		p for trend		p for interaction	
Women	< 13		13–21		22–36		≥ 37					
Cohort												
Cohort 1 (n = 1,366)	Reference		1.24 (0.93–1.67)		1.38 (1.00–1.91)		1.61 (1.08–2.39)		0.112		0.255	
Cohort 2 (n = 651)	Reference		1.15 (0.64–2.09)		1.26 (0.58–2.74)		2.48 (0.94–6.54)		0.300			
Gender												
Men (n = 916)	Reference		1.22 (0.88–1.70)		1.64 (1.11–2.40)		1.93 (1.18–3.16)		0.022		0.020	
Women (n = 1,101)	Reference		1.24 (0.82–1.89)		1.16 (0.74–1.83)		1.41 (0.81–2.45)		0.597			
Age												
< 65 years (n = 586)	Reference		2.07 (0.49–8.87)		2.06 (0.48–8.85)		2.48 (0.56–11.0)		0.670		0.299	
≥ 65 years (n = 1,431)	Reference		1.21 (0.93–1.58)		1.34 (1.00–1.80)		1.64 (1.14–2.36)		0.055			
Body mass index												
< 25 kg/m^2^ (n = 1,345)	Reference		1.26 (0.95–1.69)		1.34 (0.97–1.86)		1.90 (1.27–2.83)		0.017		0.162	
≥ 25 kg/m^2^ (n = 672)	Reference		1.22 (0.68–2.19)		1.36 (0.73–2.54)		1.46 (0.73–2.93)		0.690			
Alcohol consumption												
Non-daily drinker (n = 1,561)	Reference		1.29 (0.98–1.71)		1.21 (0.88–1.66)		1.65 (1.10–2.47)		0.087		0.181	
Daily drinker (n = 456)	Reference		1.23 (0.63–2.42)		1.98 (0.98–4.01)		1.91 (0.90–4.06)		0.043			
History of cardiovascular disease												
Absence (n = 1,802)	Reference		1.36 (1.03–1.81)		1.50 (1.09–2.06)		1.92 (1.31–2.80)		0.009		0.588	
Presence (n = 215)	Reference		1.00 (0.54–1.85)		1.00 (0.54–1.87)		1.02 (0.47–2.23)		1.00			
Antihypertensive medication												
Absence (n = 1,906)	Reference		1.36 (0.91–2.03)		1.70 (1.09–2.64)		2.92 (1.11–3.32)		0.074		0.465	
Presence (n = 1,111)	Reference		1.14 (0.80–1.61)		1.21 (0.82–1.78)		1.52 (0.93–2.49)		0.377			
Time to death												
1,095 days (n = 51)	Not examined										Not examined	
≥ 1,095 days (n = 1,966)	Reference		1.28 (0.97–1.68)		1.36 (1.01–1.84)		1.67 (1.17–2.38)		0.044			

## Discussion

The outcomes of this longitudinal study underscore the importance of GGT as a notable and autonomous indicator of all-cause mortality among individuals with hypertension residing in the community. To address concerns about reverse causality, we excluded participants who passed away during the initial three years of the follow-up period from the analysis. The exclusion of these individuals minimally impacted the outcomes. Even among individuals with GGT levels within the normal range, the correlation between elevated GGT levels and heightened mortality risk was more pronounced in men compared to women, with a statistically significant interaction observed between gender and baseline serum GGT levels (p = 0.020). As far as we know, only a limited number of studies have investigated the relationship between GGT levels within the normal range and the risk of all-cause mortality in Japanese individuals with hypertension living in community settings.

Our previous research showed that increased serum GGT levels are associated with the risk of developing hypertension [15-17], and this research revealed their association with the risk of all-cause mortality among individuals with hypertension. A meta-analysis comprising seven studies and 273,141 participants showed that the combined relative risk for all-cause mortality, comparing the highest with the lowest GGT quartile, was 1.56 (95% CI: 1.34-1.83) [18]. In a comprehensive analysis involving 35 studies with 571,511 participants and 72,196 mortality cases, it was observed that GGT, even within physiological levels, was linked to elevated all-cause mortality [10]. Increased levels of AST and GGT were significantly associated with all-cause mortality. Men with AST levels exceeding 18 U/L experienced a twofold increase in the risk of premature retirement and a threefold higher risk of all-cause mortality compared to men with lower AST levels [19,20]. Among the liver enzymes, GGT demonstrates a more pronounced and consistent positive link with heart disease, stroke, and short-term all-cause mortality compared to ALT and AST [21]. However, the magnitude of this correlation is constrained among elderly individuals and within Asian populations [18]. On the contrary, several recent studies have revealed positive correlations between GGT levels and mortality, even among elderly individuals with an average age of 70 [22,23]. Our study observed positive associations between GGT levels and all-cause mortality in the ≥ 65-year-old age group.

In this research, a notable dose-response connection was observed between GGT levels and mortality among men, with a less pronounced association noted among women. One potential rationale for this gender discrepancy is that women in our study exhibited a narrower spectrum of GGT levels within the standard deviation compared to men. As a result, there were minimal differences in risk factors across GGT tertiles among women. Nevertheless, our findings align with previous research indicating that women exhibit weaker associations between GGT levels and all-cause mortality than men [24]. Further, a health checkup cohort study has reported a stronger correlation between arterial stiffness and GGT levels in men [25]. In addition, a cohort study on the general population revealed a noteworthy correlation between changes in blood pressure and GGT levels in men, while no such association was found in women [26]. Meta-analyses revealed that the positive correlation between GGT levels and hypertension risk was more prominent in Asian and male subgroups compared to non-Asian and female subgroups [16]. These collective findings imply that GGT might serve as a predictive marker for mortality, especially among males. However, further studies are needed to investigate the underlying mechanisms behind these divergent outcomes based on gender.

There is a lack of comprehensive understanding regarding the mechanisms that contribute to the heightened all-cause mortality observed in individuals with elevated GGT levels. The hypothesis with the most robust support suggests a connection to oxidative stress [27]. The presence of active GGT in atherosclerotic plaques has been demonstrated, and it may contribute to generating reactive oxygen species during plaque development [28,29]. A longitudinal investigation unveiled a favorable correlation between GGT and inflammation indicators such as fibrinogen, C-reactive protein, and F2-isoprostanes [16]. Moreover, although lacking specificity, GGT exhibits considerable sensitivity in detecting liver damage and shows a robust correlation with both alcoholic and metabolic-associated fatty liver disease [5].

Although this study offers valuable insights into Japan's rural population, it is important to acknowledge its limitations. Initially, we utilized a cross-sectional approach, assessing baseline characteristics and GGT levels during the initial visit. However, it is important to acknowledge that GGT levels and some covariates may fluctuate over time, potentially altering during the prolonged follow-up period. As a result, the study's relevance may be underestimated because of nondiscriminatory misclassification bias, rather than overestimated. Secondly, the study relied on all deaths having been recorded, regardless of cause, in Japan's Basic Resident Register. Consequently, individuals who emigrated during the survey period may have been excluded from the analysis. Additionally, the baseline assessment encompassed various confounding factors, including medications, underlying diseases, and lifestyle adjustments, all previously associated with mortality. Despite efforts to address these confounding factors through baseline physical examinations, there could still be unmeasured factors not assessed in this study (e.g., the impact of medication on the elevation of GGT levels). Thus, further investigation is needed to explore the impact of these unexamined factors. Finally, given the relatively small participant pool and instances of mortality, this study may have underestimated the potential causal relationship between GGT levels and all-cause mortality.

## Conclusions

The research revealed a notable correlation between elevated GGT levels within the normal range and the risk of all-cause mortality among Japanese individuals with hypertension residing in community settings. This study emphasizes the necessity for future large-scale cohort investigations with prolonged follow-up durations to further explore the relationship between GGT serum levels and mortality.

## Appendices

**Table 4:** (Supplemental Table) Hazard ratios and 95% confidence intervals of baseline serum gamma-glutamyl transferase (GGT) categories for all-cause mortality in participants without hypertension Model 1 was adjusted for age and gender. Model 2 included additional adjustments for body mass index, smoking status, alcohol consumption, and history of cardiovascular disease, in addition to the covariates in Model 1. Model 3 further included adjustments for hypertriglyceridemia, hypo-HDL-cholesterolemia, hyper-LDL-cholesterolemia, diabetes, chronic kidney disease, hyperuricemia, alanine aminotransferase, and aspartate aminotransferase, in addition to the covariates in Model 2. Significant values (p < 0.05) are presented in bold.

	Baseline serum GGT categories						
Men (n = 657)	< 20	20–41	42–86	≥ 87 IU/L			
	n = 116	n = 294	n = 163	n = 84		pfor trend	
Prevalence of death, n (%)	32 (27.6)	81 (27.6)	37 (22.7)	14 (16.7)		0.171	
Model 1	Reference	1.08 (0.72–1.64)	1.30 (0.80–2.10)	1.93 (0.98–3.79)		0.219	
Model 2	Reference	1.04 (0.68–1.58)	1.33 (0.80–2.20)	2.08 (1.02–4.25)		0.140	
Model 3	Reference	1.12 (0.73–1.72)	1.36 (0.80–2.31)	2.22 (1.01–4.89)		0.231	
Women (n = 1,101)	< 13	13–21	22–36	≥ 37 IU/L			
	n = 101	n = 472	n = 217	n = 89		p for trend	
Prevalence of death, n (%)	13 (22.0)	76 (16.1)	39 (18.0)	16 (18.0)		0.682	
Model 1	Reference	0.97 (0.54–1.76)	0.85 (0.45–1.61)	1.30 (0.62–2.74)		0.561	
Model 2	Reference	0.96 (0.53–1.75)	0.83 (0.44–1.59)	1.50 (0.70–3.20)		0.308	
Model 3	Reference	1.10 (0.60–2.01)	1.00 (0.51–1.97)	2.07 (0.91–4.70)		0.597	
Overall (n = 2,017)							
Men	< 20	20–41	42–86	≥ 87 IU/L			
Women	< 13	13–21	22–36	≥ 37 IU/L			
	217	766	380	173		p for trend	
Prevalence of death, n (%)	45 (20.7)	157 (20.5)	76 (20.0)	30 (17.3)		0.813	
Model 1	Reference	1.08 (0.77–1.51)	1.10 (0.75–1.61)	1.67 (1.03–2.71)		0.162	
Model 2	Reference	1.03 (0.73–1.45)	1.08 (0.73–1.60)	1.82 (1.10–3.02)		0.055	
Model 3	Reference	1.10 (0.78–1.56)	1.15 (0.77–1.72)	2.00 (1.15–3.48)		0.065	
